# The microdomain-organizing protein MPP1 is required for insulin-stimulated activation of H-Ras

**DOI:** 10.18632/oncotarget.24847

**Published:** 2018-04-06

**Authors:** Joanna Podkalicka, Agnieszka Biernatowska, Paulina Olszewska, Sabina Tabaczar, Aleksander F. Sikorski

**Affiliations:** ^1^ Laboratory of Cytobiochemistry, Faculty of Biotechnology, University of Wrocław, 50–383 Wrocław, Poland

**Keywords:** small GTPases, H-Ras, PM-lateral organization, MPP1

## Abstract

Signaling complexes are localized to distinct plasma-membrane domains which undergo precise spatiotemporal regulation. A crucial link between membrane dynamics and the small GTPase, H-Ras, has been suggested, connecting membrane localization, clustering and scaffolding with its activity and signal transduction. Results of this study suggest a relationship between MPP1 and/or MPP1-dependent plasma-membrane organization and H-Ras activation. Namely, we show here that in HEL cells, *MPP1* knock-down lead to the disruption of signaling cascade(s) from the activated insulin receptor. The signal inhibition occurred at the level of H-Ras, as it showed impaired GDP-to-GTP exchange and further interaction with its effector molecule, Raf. Moreover, in these cells H-Ras detergent-resistant membrane localization was not sensitive to insulin treatment which may imply molecular mechanism via which MPP1 affects functions of other proteins which may be connected with functional domain formation. Understanding the link between MPP1 and activation of H-Ras, may provide an important insight into the complexity of Ras related signaling pathways which may become a potential target for associated cancer therapies.

## INTRODUCTION

The plasma membrane (PM) constitutes a complex environment, laterally subdivided into highly dynamic domains, existing on different temporal and spatial scales, which provide platforms for the assembly of many signal transduction pathways [1]. Ras GTPases are PM-localized molecular switches that regulate multiple signaling cascades involved in proliferation, differentiation, and cell survival [2]. Aberrant functions of Ras proteins have been known for many years to be correlated with many types of human cancer and a number of developmental disorders [3–5] what triggered many attempts to find Ras inhibitor as potential therapeutic compounds [6]. Nevertheless, due to the lack of deep binding pockets in Ras protein, finding a small molecule which binds Ras with high affinity has been so far unsuccessful (reviewed in [7]). Moreover, as Ras proteins are essential for signal transduction from receptor tyrosine kinases (RTKs) proposed inhibitor should be specific towards distinct isoform as inhibition of all Ras isoforms is expected to be highly toxic. Thereby, there is still justified need for a novel therapeutic approach that would target Ras activation and subsequent signaling pathways.

There are four Ras protein isoforms ubiquitous in human cells, H-, N- and two forms of K-Ras (4A and 4B), generated through alternative splicing of the fourth coding exon, all of which need to be anchored to the PM through their lipid-modified C-terminus, for proper function [8, 9]. All isoforms possess a CAAX S-farnesylation motif, with one (N-Ras) or two (H-Ras) additional S-palmitoylation sites or polybasic region (K-Ras) [10–13]. Based primarily on differences in lipidation patterns, the affinity of Ras isoforms to cholesterol-enriched PM-domains is diverse, leading to the formation of distinct, non-overlapping Ras nanoclusters, which are thought to be responsible for the observed functional divergence among Ras isoforms [14–16]. The highest affinity for liquid ordered (l_o_) domains is observed for GDP-bound H-Ras, with a decreased affinity for its activated, GTP-bound form, and which is undetectable for both the active and inactive forms of K-Ras [16–19]. Apart from differential association of Ras isoforms with distinct PM domains, activation of Ras effectors is also influenced by a local lipid composition within the membrane [20].

Despite the fact that we are getting more insights into PM heterogeneity and structure, a precise molecular mechanism underlying membrane-domain formation in natural membranes is still missing. It is well established that PM dynamics play a crucial role in cancer progression and metastasis by regulating the activity of many membrane receptors, adhesion molecules as well as extra cellular matrix (ECM) remodeling proteins (reviewed e.g. in [21]). Our recent studies demonstrated that a peripheral membrane protein, MPP1/p55 (membrane palmitoylated protein 1), takes part in the regulation of PM lateral organization of erythrocyte precursors (reviewed in [22]). MPP1, a MAGUK (membrane associated guanylate kinases) family protein, was originally described in erythrocytes as a skeletal protein that participates in the linking of the membrane skeleton elements to the PM by formation of a tripartite complex with protein 4.1 and glycophorin C [23–25]. It has also been suggested that MPP1 regulates membrane polarity of chemoattractant-activated neutrophils, by the accumulation of PI(3,4,5)P_3_ at the leading edge of migrating cells and downstream Akt phosphorylation [26]. Our further studies revealed that both MPP1 protein depletion and the inhibition of its palmitoylation reduced the relative order of the PM in erythrocyte precursors and PM-derived vesicles possibly through disruption of domains formation, suggesting that these changes could affect signal transduction from membrane-bound growth factor receptors [27, 28].

Here we demonstrate that mechanisms behind PM spatiotemporal organization generate an additional level of cell-signaling networks regulation, linking MPP1 and possibly MPP1-dependent regulation of membrane dynamics with activation of domain-related H-Ras. These findings provide an important insight into the complexity of Ras related signaling pathways which can become a potential target for associated cancer therapies.

## RESULTS

### Decreased level of MPP1 causes impaired signal-transduction from activated IR

**Figure 1:**
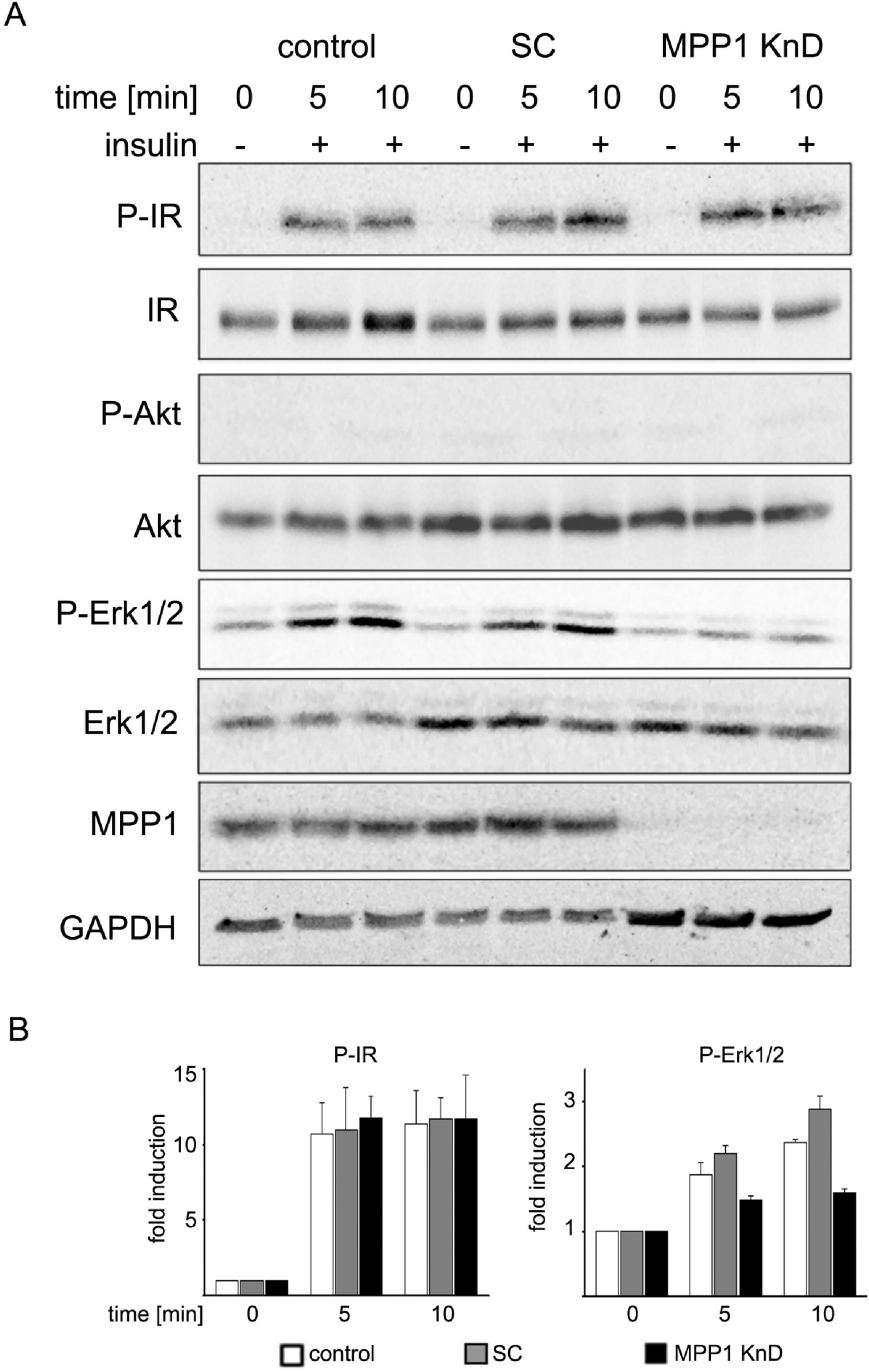
*MPP1* knock-down inhibits signal transduction from activated IR (**A**) Control, SC and MPP1 KnD cells were treated with insulin [1 μg/ml] at the indicated time points, after which the whole-cell extracts were subjected to Western Blot analysis and probed with the indicated antibodies. GAPDH was used as loading control. (**B**) Quantification of the relative phosphorylation levels of IR and Erk1/2 in control, SC and MPP1 KnD cells (average ± S.D. from three independent experiments).

We have recently shown that a specific molecule, MPP1, affects PM fluidity and domain formation in erythroid cells (see Introduction). To assess the functional role of MPP1 in membrane dynamics, we used a stable, lentiviraly transduced, *MPP1* knock-down human erythroleukemia cell line (MPP1 KnD) along with control cells: wild-type HEL (WT, control) and HEL transduced with scrambled shRNA (SC) (Supplementary Figure 1A). Activation of the insulin receptor (IR) was chosen as a model signaling pathway, as IR is highly abundant in the PMs of the analyzed cell lines (Supplementary Figure 1A) and it was previously shown that membrane dynamics plays an important role in IR signaling [29, 30]. Saturated IR and downstream extracellular signal-regulated kinases (Erk1/2) activation was observed in control cells between 1 to 60 minutes after stimulation with insulin (Supplementary Figure 1B, 1C, also [27]). Interestingly, MPP1 KnD cells showed the same level of IR phosphorylation in comparison to both mentioned above controls. However, signal transduction was impaired in MPP1 KnD cells, as observed by markedly decreased phosphorylation of Erk1/2 (Figure 1A), which was similar to the level of non-stimulated cells, whereas SC and WT cells showed approximately a two to three-fold Erk1/2 activation upon insulin treatment (Figure 1B). To test whether *MPP1* silencing directly affects intracellular kinases, cells were stimulated with 100 nM phorbol ester (PMA) as a control. No significant differences in the activation of Akt and Erk1/2 were noticed between MPP1 KnD and both controls (Supplementary Figure 2).

### Erk1/2 signaling cascade is interrupted in insulin-treated MPP1 KnD cells

**Figure 2:**
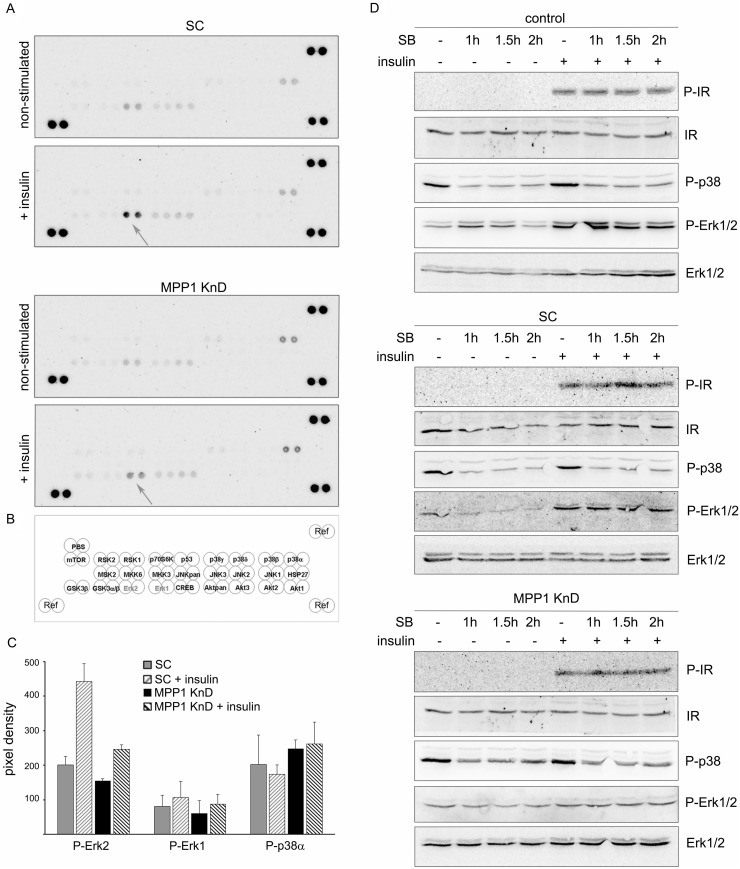
Only the Erk1/2 signaling cascade downstream of the activated IR is interrupted in MPP1 KnD cells (**A**) SC (top) and MPP1 KnD cells (bottom) were treated or not treated, with insulin [1 μg/ml] for 10 min, after which whole-cell extracts were subjected to kinase activation analysis. (**B**) Schematic representation of activated kinases membrane spots. (**C**) Quantification of the relative phosphorylation levels of Erk1, Erk2 and p38α in SC and MPP1 KnD cells normalized to reference spots (marked as ‘Ref’) (average ± S.D. from four independent measurements). (**D**) Control, SC and MPP1 KnD cells were pretreated with SB202190 [10 μM] for 1, 1.5 and 2 h before insulin treatment for 10 min. Whole-cell extracts were subjected to Western Blot analysis and probed with the indicated antibodies.

To answer the question why signal transduction is inhibited, we first tested which pathways are affected in MPP1 KnD cells stimulated with insulin. MPP1 KnD and SC cells were treated with insulin and subsequently the whole-cell lysates were subjected to a kinase activation screen in which the phosphorylation level of 26 different kinases was determined. Interestingly, the only activated kinase which was detected in SC cells was Erk2 and we did not detect the activation of any other kinase, including Akt, for either of the two cell lines (Figure 2A top, 2B, 2C). Once again, we observed that MPP1 KnD cells showed much lower phosphorylation of Erk2 kinase than SC cells (Figure 2A bottom, 2B, 2C). Interestingly, in some cell preparations we also observed an increased level of phosphorylated p38α in MPP1 KnD cells relative to the SC cells, independent of insulin treatment. However, this change appeared statistically insignificant (Figure 2C). Zhang and coworkers have shown that up-regulation of p38α could inhibit Erk1/2 activation by the direct interaction of those two kinases resulting in sequestration of Erk1/2 [31]. In order to test this hypothesis, we used SB202190, a specific p38 MAP kinase inhibitor, in insulin-treated cells. We did not observe any recovery of Erk1/2 phosphorylation in MPP1 KnD cells, along with no particular change in the Erk phosphorylation pattern in controls after inhibition of p38 phosphorylation (Figure 2D) suggesting that the inhibition of Erk1/2 activation in cells is not due to the increased activity of p38α.

### H-Ras activation depends on MPP1 in insulin-treated erythroid cells

**Figure 3:**
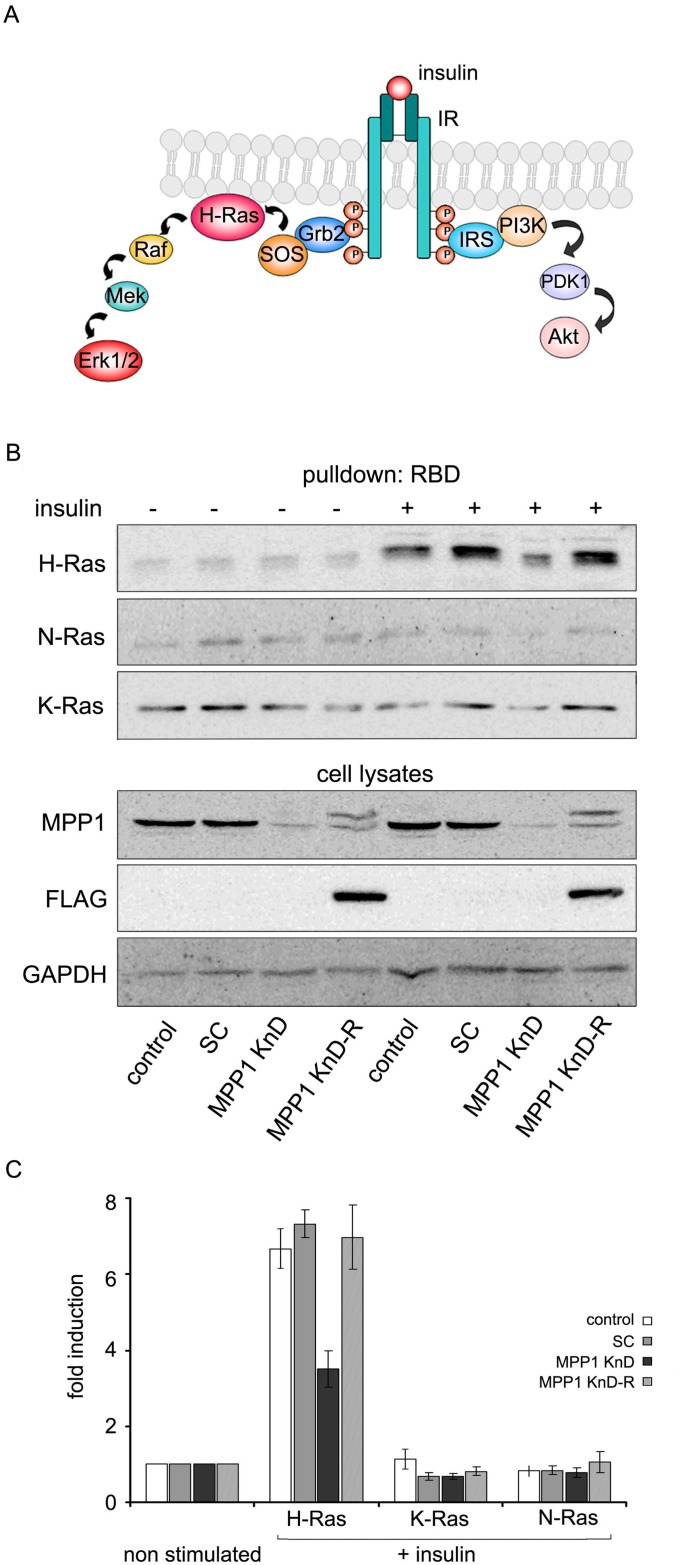
Signal transduction from activated IR is arrested at the level of H-Ras (**A**) Schematic diagram of two alternative signaling pathways downstream of activated IR. (**B**) Control, SC, MPP1 KnD and MPP1 KnD-R (KnD cells transfected with rescue vector encoding FLAG-tagged MPP1) cells were treated with insulin [1 μg/ml] for 5 min, after which 600 μg of total protein from whole-cell extracts were subjected to pull-down of active H, K and N-Ras-GTP using GST-Raf1-RBD. Additionally, the same whole-cell extracts were probed with the indicated antibodies. The double band and positive reactivity with anti-FLAG antibodies comes from expression of FLAG-tagged MPP1 from rescue mutant. Note: due to close values of mol. wt. data for GAPDH and FLAG are coming from the same samples but from different gels. (**C**) Quantification of the levels of activated H-, K- and N-Ras-GTP normalized to GAPDH levels (average ± S.D. from three independent measurements). RBD–Ras binding domain, WB–Western blot.

Activation of Erk1/2 downstream of IR requires recruitment of the Grb2 adaptor molecule and subsequent activation of Ras via SOS-mediated GDP-to-GTP nucleotide exchange (Figure 3A). We tested Ras activation in insulin-treated MPP1 KnD cells. For this purpose, a pull-down assay was used in which whole-cell lysates were loaded on a resin pre-coupled with Raf-1-derived Ras-binding domain (RBD). The activation of the three Ras isoforms, H, K and N-Ras was checked. For both control and SC cells, insulin-stimulation resulted in potent H-Ras activation, while approximately three times lower H-Ras-GTP level was detected in the MPP1 KnD cells. The other two Ras isoforms were not activated in any of the analyzed cell lines (Figure 3B, 3C). Moreover, to validate that the observed effects can be restored, and are not a result of the off-target effect of *MPP1*-gene silencing, we performed a recomplementation experiment in MPP1-KnD cells by introducing mutated FLAG-MPP1 that is resistant to shRNA, as described before [28]. Cells with restored expression of MPP1 (MPP1 KnD-R) and stimulated with insulin were characterized by H-Ras activation reaching the level of controls (Figure 3B, 3C).

**Figure 4:**
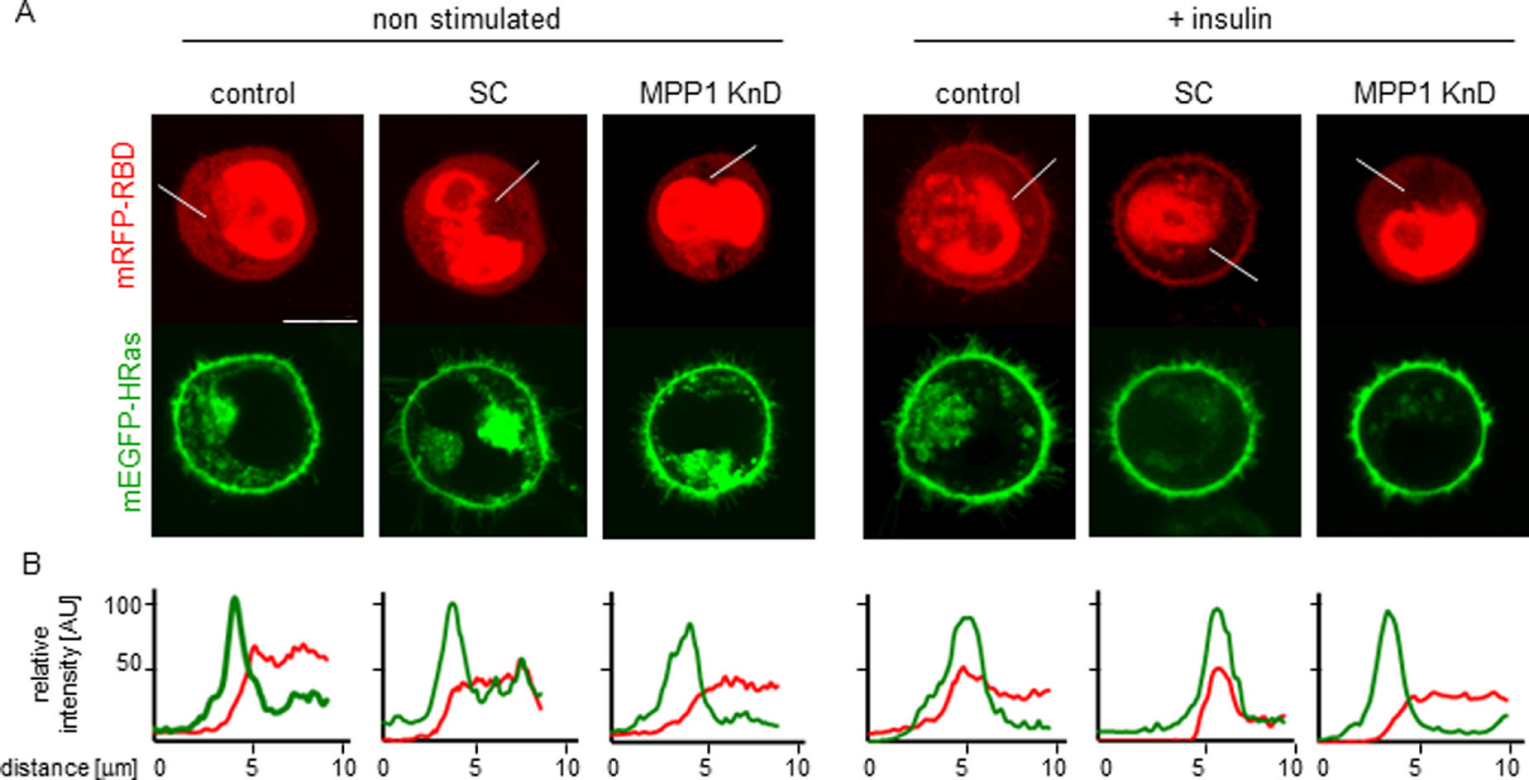
Localization of mEGFP-HRas and mRFP-RBD in non-stimulated and insulin treated cells (**A**) Representative images, scale bar 10 um, (**B**) Relative fluorescence intensity profiles.

**Figure 5:**
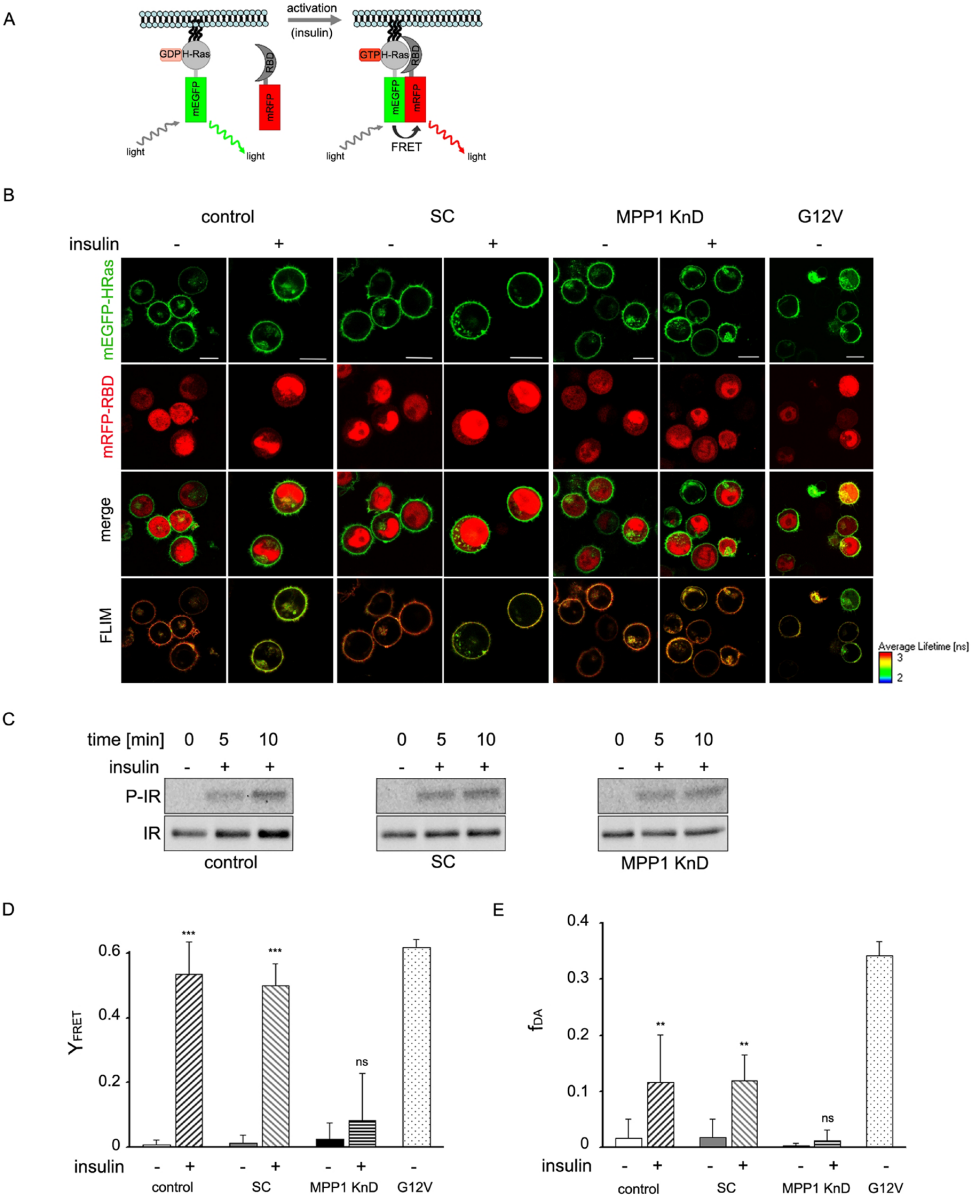
H-Ras activation depends on MPP1 (**A**) Schematic representation of FRET between activated mEGFP-HRas and mRFP-RBD. (**B**) Exemplary confocal and FLIM-FRET images of control, SC and MPP1 KnD cells overexpressing mEGFP-HRas and mRFP-RBD, treated or not treated with insulin [1 μg/ml]. FLIM image color look-up table on the bottom-right represents fluorescence lifetime. G12V was used as the FRET positive control. Scale bars 10 μm. (**C**) Cells subjected to FLIM-FRET analysis were collected and whole-cell extracts were analyzed by Western Blot with the indicated antibodies. (**D**) The Y_FRET_ was calculated for cells treated with insulin [1 μg/ml] according to equation 1 (see Material and Methods). The values depict the mean ± S.E.M. Three independent biological repeats were analyzed. Statistical significance was determined by one way ANOVA with a post hoc Tukey-Kramer test, and differences were considered statistically significant at *p* < 0.01 (^**^), *p* < 0.001 (^***^), ns – not significant (*p* > 0.05). (**E**) Fraction of donor bound to acceptor (f_DA_) after treatment with insulin. The values represent mean ± S.E.M. Statistical significance was determined as in (D). See also Supplementary Table 1. All analyses performed on cells with optimal relative fluorescence intensities (see Supplementary Figure 4).

To confirm changes in H-Ras activity we analyzed direct interactions of H-Ras with its effector molecule, Raf, via another methods. First we analyzed membrane recruitment of cRaf to activated H-Ras (Figure 4A). We observed change of localization of RBD domain of cRaf towards PM upon insulin treatment in control and SC cells while in KnD cells no change was detected (Figure 4A). Detailed analysis of localization revealed colocalization with H-Ras (Figure 4B). Additionally we performed Förster resonance energy transfer (FRET) between mEGFP-HRas (donor) and mRFP-RBD from cRaf (acceptor) overexpressed in control, SC and MPP1 KnD cells treated with insulin (Figure 5A), to follow direct interaction between H-Ras and RBD. We also used the mEGFP-labeled constitutively active H-Ras mutant (G12V), which has a high affinity for RBD [32], as a positive control of H-Ras activity. FRET provides information about direct interaction between molecules, as its efficiency highly depends on the distance between them and is observed only at nanometer-range proximity [33]. One of the methods to quantify FRET efficiency is the measurement of the fluorescence-lifetime of a donor, in the presence or absence of acceptor, by using fluorescence lifetime microscopy (FLIM). This method allows the resolution of both the fractional contribution and fluorescence lifetimes of interacting and non-interacting species. Therefore, to examine the interaction between activated H-Ras and RBD, we used FLIM-FRET methodology. Cellular localization and fluorescence lifetimes of both donors only (mEGFP-HRas and G12V) were determined (Supplementary Figure 3). The obtained lifetime values were at 2.50 ± 0.01 and 2.47 ± 0.02 ns respectively. FLIM-FRET images showed an increased FRET after insulin stimulation of both controls, observed as significantly decreased mean mEGFP-HRas fluorescence-lifetimes, while almost no lifetime-change was observed for insulin-treated MPP1 KnD cells (Figure 5B). To confirm IR activation, samples were collected and analyzed by Western Blot. All cell-lines showed comparable IR phosphorylation levels (Figure 5C), indicating that all cells were activated. After fitting to a bi-exponential decay model, FRET efficiencies were calculated for each donor-acceptor pair relative to the donor only. Apparent FRET efficiency (Y_FRET_) markedly increased for control and SC cells treated with insulin (0.52 ± 0.1, 0.49 ± 0.08 respectively), reaching the level comparable with constitutively active G12V (0.53 ± 0.03), while no significant increase in Y_FRET_ was observed for insulin-treated MPP1 KnD cells (0.08 ± 0.14) (Figure 5D). Moreover, the contribution of the bound donor-acceptor pair (f_DA_) for both controls increased after addition of insulin (0.12 ± 0.08 control, 0.12 ± 0.05 SC) while, for MPP1 KnD, there was no substantial increase in the binding fraction (0.01 ± 0.02) (Figure 5E). However, the contribution of the binding fraction detected for controls was relatively low, compared to G12V (0.34 ± 0.03). This can be explained by the fact that only a fraction of mEGFP-HRas was activated in the insulin-treated cells while majority of G12V is constitutively active. Binding fraction reflects more precisely the actual situation as it dissects the contribution of distinct fluorescence species. All the data, including mean fluorescence lifetimes and the χ^2^ value, which describes parameters of fitting, are summarized in Supplementary Table 1.

H-Ras lateral segregation into different types of nanoclusters in the PM highly depends on its activation state [11, 34]. GDP-bound H-Ras resides predominantly in l_o_ membranes, while its GTP-loaded form is released into the l_d_ part of the membrane, followed by clustering with galectin-1 [35–37]. Based on our previous studies, which demonstrated that MPP1 affects the lateral organization of the PM, we decided to analyze H-Ras PM localization in the context of MPP1 depletion. For this purpose, detergent-resistant membrane (DRM) flotation in a sucrose gradient was used (described previously by others [38]) and DRM fraction from insulin-treated, mEGFP-HRas transfected cells were examined for H-Ras localization. We observed that, for both controls, insulin stimulation resulted in H-Ras shift from the DRM towards soluble fractions, while in MPP1 KnD cells, we did not observe any change in H-Ras localization in the gradient profile (Supplementary Figure 5). DRM isolation constitutes highly controversial method and is not sufficient to conclude on membrane dynamics, however, even in this harsh conditions we could still observe differences in H-Ras membrane association, what strengthens the role of MPP1 in control of H-Ras membrane localization/dynamics.

In conclusion, the data presented above suggests that an impairment of Erk1/2 signaling in MPP1 KnD erythroid cells may result from disturbed H-Ras activation which could be linked to the decreased MPP1 level and possibly to changes in PM lateral organization of these cells.

## DISCUSSION

Currently, membrane-rafts [39] are defined as dynamic, sterol-sphingolipid-enriched, ordered nanoscale assemblies of proteins and lipids, with a lipid structure that is equivalent to the l_o_ phase of model membranes [1]. One of their pivotal roles is spatio-temporal separation of different signaling events occurring at the PM. Many of the membrane receptors, as well as adaptor and signaling molecules localize at PM domains, including the group of Ras small GTPases [40]. As demonstrated by numerous independent studies, Ras isoforms form distinct nanoclusters and their activation is highly dependent on their PM localization (for review see [14]). Based on these findings, one cannot overestimate the role of the mechanisms underlying PM lateral dynamics in the regulation of cell-signaling networks and Ras-mediated cancer development. However, only limited data is available on biological mechanisms responsible for their assembly in natural membranes. It was proposed that small (<10 nm), short lived (<0.1 ms) l_o_ nanoclusters, “precursor” domains, form spontaneously in the PM and can be stabilized by lipid-anchored or transmembrane proteins, leading to their coalescence into larger assemblies which could then be considered as functional rafts [41]. We have previously demonstrated that one of such a domain-triggering lipid-anchored protein could be MPP1, which regulates membrane-order in erythrocyte precursors [27, 28]. In this study we show that MPP1-dependent membrane dynamics, in fact, may constitute a regulatory mechanism of functional membrane-domain formation as it is crucial for proper signal-transduction downstream of activated IR. Cells with a decreased level of MPP1, display decreased Erk1/2 phosphorylation upon insulin treatment (Figure 1A, 1B) independent on direct influence on MAPK (Supplementary Figure 2). Signal arrest appears at the level of H-Ras as we observed impaired GDP-to-GTP exchange. This would be in agreement with data of e.g. Zhang and Lodish [42]. The fact that it was restored after MPP1 recomplementation in KnD cells (Figure 3B) strongly supports the role of MPP1 at this stage of the mentioned signaling pathway. No interaction of H-Ras with its effector molecule, Raf, was observed, shown as no change in RBD localization (Figure 4) and a markedly decreased FRET efficiency between mEGFP-HRas and mRFP-RBD (Figure 5). To check whether the effect of MPP1 depletion could also arise from the possibility of MPP1 to have the activity of H-Ras GEF or GAP, *in vitro* GEF/GAP activity assays were performed, however neither GEF nor GAP activity of recombinant MPP1 was observed (Supplementary Figure 6).

Different Ras isoforms exhibit distinct plasma membrane localization, dependent on GDP or GTP loading. H-RasGDP predominantly resides in the l_o_ domains of the PM, while upon nucleotide exchange to H-RasGTP it dissociates from l_o_ domains and forms transient nanoclusters with Gal-1, which decreases H-Ras mobility [42]. We have shown here that upon insulin treatment H-Ras remains within DRM fraction of MPP1 KnD cells (Supplementary Figure 5), suggesting that the loss of an appropriate initial lateral PM-organization may be connected to the impairment of H-Ras functionality.

Our latest data shows that MPP1 triggers domain-assembly through interactions with flotillins, which are well-defined raft marker proteins [43]. These findings, along with the data presented here, are in line with a recent study which shows that flotillin-1 is required for epidermal growth factor-induced activation of H-Ras in breast cancer cells [44]. The authors speculate that deficiency of flotillin-1 may alter the structure of PM domains, thereby affecting the localization and activation of H-Ras. Our data seem to support and provide an explanation of these observations.

Mutations in RAS genes have been known to play a causal role in cancer development and are associated with approximately one million deaths per year worldwide. Though, targeting Ras signaling appears to be attractive and justified approach for novel cancer therapies. Nevertheless, unique properties of Ras proteins rise major challenges that need to be overcome, like mentioned before lack of deep binding pocket that could be recognized by possible drugs. As Ras activation and its interaction with both upstream and downstream molecules require proper membrane association, understanding PM dynamics could be crucial for future drug development. Both GEFs and GAPs which turn Ras on and off need to be recruited to the PM. Also Raf kinase, effector molecule activated by Ras, needs to be recruited to the PM sites displaying distinct lipid specificity [45] and it is a critical step for further signaling.

The data presented here introduce MPP1 as a molecule crucial for the regulation of signal transduction, through the modulation of PM spatio-temporal organization. We demonstrate the link between MPP1-dependent PM dynamics and activation of PM-bound H-Ras, comprising a piece of the puzzle in the understanding of the complexity of membrane-associated signal-transduction pathways which could comprise a new target for future cancer therapies.

## MATERIALS AND METHODS

### Cell culture

Cells were grown in RPMI 1640 medium supplemented with 10% FBS, 2 mM glutamine, 100 units/mL penicillin, and 100 μg/mL streptomycin at 37° C in a humidified atmosphere of 5% CO_2_. MPP1 KnD and SC cultures were supplemented with 2 μg/mL of puromycin. *MPP1* gene silencing was performed as described before [27].

### Plasmids and antibodies

mEGFP-HRas, mEGFP-HRasG12V and mRFP-RBD(R59A)-mRFP plasmids were a gift from Karel Svoboda (Addgene plasmids respectively: #18662, #18666, #18664) [46]. FLAG-MPP1 for MPP1 recomplementation was described before [27]. Anti-MPP1 antibodies were purchased from Abnova, anti-GAPDH (Abcam), anti-FLAG (Sigma-Aldrich), anti-P-IGFIRβ(Tyr1135/1136)/IRβ(Tyr1150/1151), anti-IRβ, anti-P-Erk1/2(Thr202/Tyr204), anti-Erk1/2, anti-P-Akt(Ser473), anti-Akt, anti-P-p38(Thr180/Tyr182) and active Ras Detection Kit (Cell Signaling Technology), anti-HRas antibodies (GeneTex), anti-N and KRas antibodies (Aviva Systems Biology), Human Phospho-MAPK Array (R&D Systems), anti-GFP, Secondary antibodies conjugated with HRP (SantaCruz), Cholera Toxin β conjugated with HRP (Life Technologies).

### MAPK activity array, active H-Ras pull-down and immunoblotting

Cells were seeded at 2 * 10^5^ (immunoblotting), 1 * 10^6^ (phospho-MAPK array) or 5 * 10^6^ (active H-Ras pull-down) and serum-starved for 20 hours before treatment with human recombinant insulin (Sigma-Aldrich) [1 μg/ml] at the indicated time points. For immunoblotting, the stimulated cells were harvested, washed with ice-cold PBS and lysed for 30 minutes on ice in the lysis buffer (50 mM HEPES, pH 7.5, 100 mM NaCl, 1 mM EDTA, 10% glycerol, 0.5% NP-40) supplemented with 100 μM PMSF, protease inhibitor cocktail (Sigma-Aldrich) and phosphatase inhibitors cocktail (SantaCruz). Proteins were separated by SDS-PAGE, followed by Western Blot analysis. All immunoblotting data presented here is representative from three independent experiments. In the case of the phospho-MAPK array and active H-Ras pull-down, all steps were performed according to the manufacturers’ protocols. In brief, after stimulation with insulin, cells were washed once with ice-cold PBS and lysed for 30 minutes and 10 minutes, respectively, on ice in lysis buffers supplied by manufacturer. Extracts were clarified by centrifugation and protein concentration was determined using BCA. 200 μg of total protein from whole-cell extracts were subjected to MAPK activation analysis and 600 μg for active H-Ras pull-down. For the MAPK array, the supplied biotinylated detection antibodies, in combination with streptavidin-HRP, were used for immunoblotting. Membranes were analyzed according to the template. Outermost spots served as positive and PBS spots as negative controls. For the H-Ras activation assay, recombinant GST-Raf1-RBD (Ras binding domain) was added to glutathione resin along with cell lysates. After 1 hour of incubation with the resin at 4° C, samples were washed three times with lysis buffer and subsequently released from the resin by addition of sample buffer. Proteins were separated by SDS-PAGE, followed by Western Blot analysis. The activated Ras isoforms were detected with dedicated antibodies. Total lysates (30 μg), the same as used for pull-down assay, were separated and immunoblotted for MPP1, Flag and GAPDH. Blots were quantified and normalized to appropriate loading controls.

### FLIM-FRET analysis of mEGFP-HRas and mRFP-RBD

1 * 10^6^ cells were transfected with the appropriate plasmids (0.5 μg each) using CLB (Lonza) electroporation. After 24 hours, cells were serum-starved for 20 hours and transferred onto glass coverslips coated with 0.1 mg/ml poly-L-lysine. Cells were observed in a chamber providing 37° C and 5% CO_2_. Images were taken before and after addition of insulin [1 μg/ml], 1–10 min after stimulation. Confocal images of mEGFP-HRas and mRFP-RBD were obtained using a LSM 510 META microscope (Carl Zeiss, GmbH, Germany). Samples were excited at 488 nm for mEGFP and 561 nm for mRFP and imaged with an 40× WI objective (NA 1.2), using a BP 505–530 and LP 575 filters for mEGFP and mRFP respectively. For FLIM measurements, only cells with optimal relative fluorescence intensities of both mEGFP-HRas and mRFP-RBD ranging between 100–200 A.U., were analyzed (Supplementary Figure 4). FLIM-FRET measurements were acquired by time-correlated single-photon counting (TCSPC) with the same microscope equipped with FLIM dedicated optics from PicoQuant. Samples were excited at 470 nm and imaged using a BP 503–521 filter set. Acquisition time was adjusted to collect at least 1,000 photons per pixel. Laser power was adjusted to achieve a photon collection not exceeding 900 photons/s. Images were acquired in a 256 × 256 format, and the fluorescence lifetime was calculated for each pixel. Data acquisition and processing was conducted using the SymPhoTime software (PicoQuant). Each pixel in the image was pseudo-colored according to the average fluorescence-lifetime. FLIM-FRET data was analyzed, after convolution of fluorescence decay and the instrument response function (IRF) of the FLIM system, by two fluorescence decay models, where each was validated by weight residuals and the χ^2^ value, which is close to 1 for a good fit. All decays were first fitted to a single exponential model, with those data that did not fit this model fitted to a bi-exponential decay model according to the equation 1: I(t) = A_1_exp^(−t/τ1)^ + A_2_exp^(−t/τ2)^, with τ_1_ fixed to value obtained for donor alone (2.5 ns), where τ stands for fluorescence lifetime and A for amplitude of individual τ contribution. The apparent FRET efficiency was calculated according to the equation 2: Y_FRET_ = 1 – τ_DA_/τ_D_, using measured lifetimes of each donor-acceptor pair (τ_DA_) and the average lifetime of the donor only (τ_D_). Measurements and the data analysis were performed according to precise TCSPC protocol published before [47]. Obtained FRET efficiencies were referenced against the mEGFP-HRasG12V mRFP-RBD donor-acceptor pair, as positive control. The binding fraction (f_DA_) was calculated from the contribution of each fraction of the donor molecules, namely, interacting and non-interacting components, based on the amplitudes of each individual contribution.

## SUPPLEMENTARY MATERIALS FIGURES AND TABLES


