# Supplementary material for: The microdomain-organizing protein MPP1 is required for insulin-stimulated activation of H-Ras
Source: Oncotarget. 2018 Apr 6;9(26):18410–21. doi: 10.18632/oncotarget.24847 (PMC5915081; doi:10.18632/oncotarget.24847)
Supplement: Supplementary file 1 [file oncotarget-09-18410-s001.pdf]

The microdomain-organizing protein MPP1 is required for insulin-stimulated activation of H-Ras

SUPPLEMENTARY MATERIALS

DRM isolation

1 * 10^7 cells were transfected with mEGFP-HRas plasmid (4 µg) using CLB (Lonza) electroporation. After 24 h, cells were serum-starved for 20 h before treatment with insulin [1 µg/ml] for 5 min and subsequently collected and treated as described before [38]. In brief, cells were collected by centrifugation and washed twice in ice-cold PBS. Subsequently, cell pellets were resuspended in 0.5 ml of 25 mM Tris (pH 7.4), 150 mM NaCl, 5 mM EDTA, and 0.25% Triton X-100, supplemented with a protease inhibitor cocktail (Sigma-Aldrich), and incubated at 4° C for 1 h on a rocker. Lysates were adjusted to a sucrose concentration of 45% by addition of 0.7 ml of 77% sucrose in 10 mM Tris (pH 7.4). Next, 2 ml of 35% sucrose and 1 ml of 16% sucrose were sequentially overlaid on top and centrifuged in a Beckman SW60Ti rotor for 18 hours at 38 500 rpm. Nine fractions were collected from the top of the gradient (470 µl each). A 2 µl aliquot of each fraction was taken for dot-blot analysis with cholera toxin β HRP (CTxB), to detect the DRM fraction. The remaining part of each fraction was precipitated in 10% trichloroacetic acid, resuspended in loading buffer and separated by SDS-PAGE, followed by Western Blot.

Recombinant MPP1 preparation

For *in vitro* GEF/GAP activity assay recombinant MPP1-GST was expressed in *E. coli* strain BL21DE3, immobilized on glutathione-Sepharose 4B beads (GE Healthcare) and purified in the native conditions (10 mM HEPES pH 7.4, 150 mM NaCl buffer). GST-tag was cleaved off on the column with the PreScission protease (Sigma) according to the manufacturer's protocol. Purified untagged MPP1 was dialyzed against 150 mM NaCl solution and subsequently used for GEF or GAP activity assay.

GEF activity assay

In vitro GEF activity assay was prepared by using RhoGEF Exchange Assay Biochem Kit (Cytoskeleton, Inc.) according to the manufacturer's instructions. Fluorescence spectroscopic analysis of *N*-methylanthraniloyl (mant)-GTP incorporation into small GTPases (His-H-Ras, His-RhoA, His-Cdc42) was carried out using Carry Eclipse spectrofluorimeter at 20° C. Mixtures were prepared in black 96 half well plate and fluorescence measurements were taken approximately every 30 second with excitation and emission wavelengths of 360 nm and 440 nm, respectively. After five readings (150 sec), purified MPP1 protein (1 and 0.6 µM) or hDbs (0.8 µM) were added to appropriate wells, and the relative mant-GTP fluorescence was monitored for 30 minutes. The GEF activity of hDbs on Cdc42 or RhoA GTPases was used as positive controls whereas negative control was prepared by using His-H-Ras protein only.

Rho/GAP assay

In vitro GAP activity assay was performed using RhoGAP Assay Biochem Kit (Cytoskeleton, Inc.). Briefly, purified MPP1 (2 and 3 µM) was incubated with 6 µM His-tagged H-Ras (Cytoskeleton) and 200 µM GTP (Cytoskeleton) with or without RhoGAP domain at 37° C for 20 min. Free phosphate obtained during reaction was measured by addition of CytoPhos reagent (Cytoskeleton), and the absorbance was subsequently monitored at 650 nm. The GAP activity of RhoGAP on Cdc42, RhoA or Rac 1 GTPases was used as a positive controls. Negative controls were performed without addition of RhoGAP domain in reaction mixture.

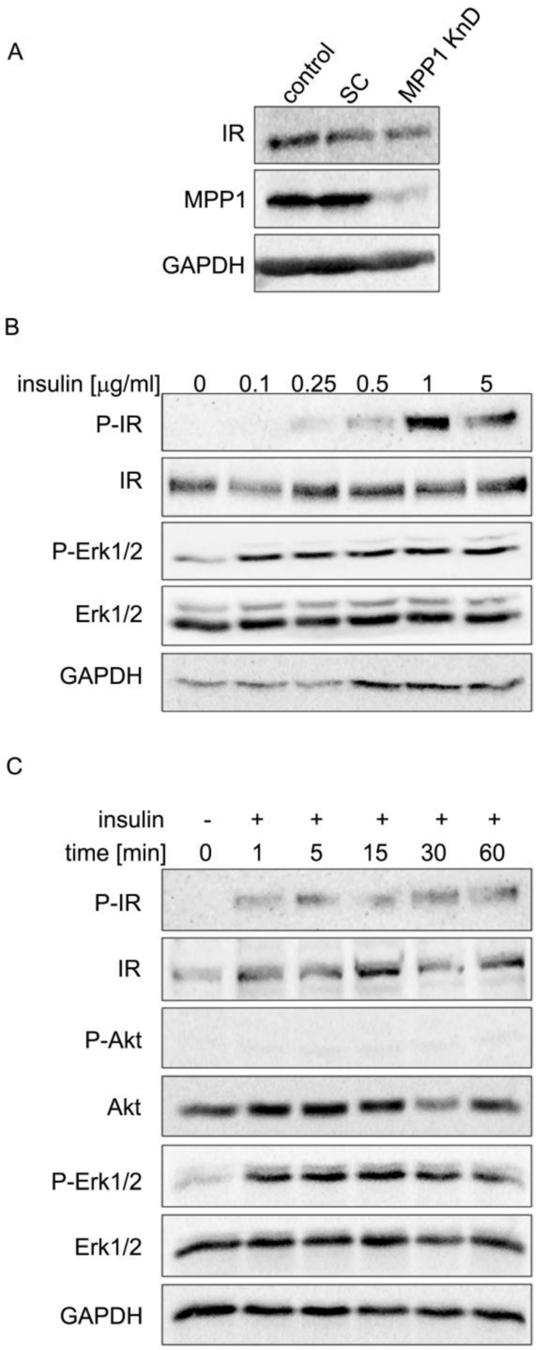

Supplementary Figure 1: (**A**) Western Blot analysis showing the level of IR (upper panel) and MPP1 (middle panel) in control, SC and MPP1 KnD cells. (**B** and **C**) Control cells were treated for 15 minutes with insulin in increasing doses, as indicated (B), or with a fixed insulin concentration of 1 μg/ml at the indicated time points (C). Subsequently, whole-cell extracts were subjected to Western Blot analysis and probed with the indicated antibodies. In all cases, GAPDH was used as loading control.

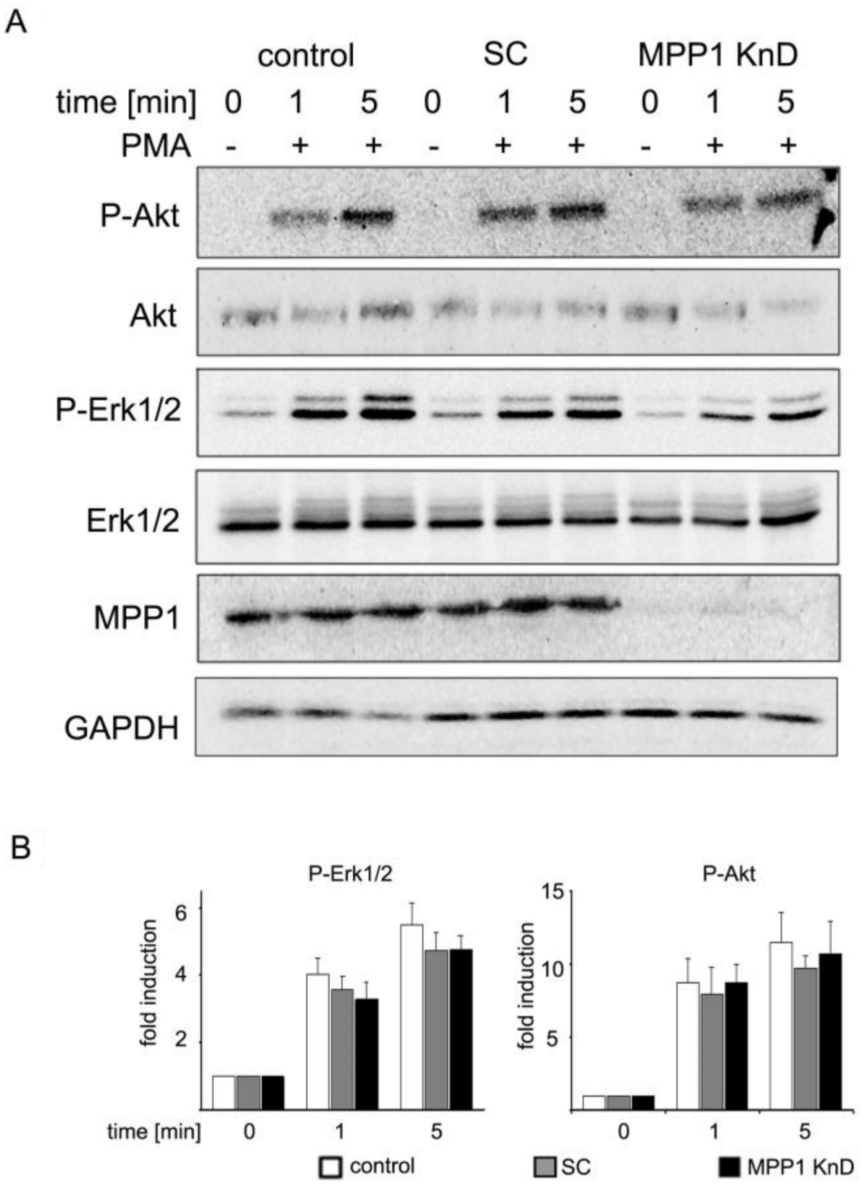

Supplementary Figure 2: (**A**) Control, SC and MPP1 KnD cells were treated with PMA [100 nM] as a control of activation and analyzed by Western Blot with the indicated antibodies. GAPDH was used as loading control. (**B**) Quantification of the relative phosphorylation levels of Erk1/2 and Akt in control, SC and MPP1 KnD cells (average ± S.D. from three independent experiments). Both quantifications were normalized to GAPDH levels. Representative immunoblotting data from three independent experiments are presented.

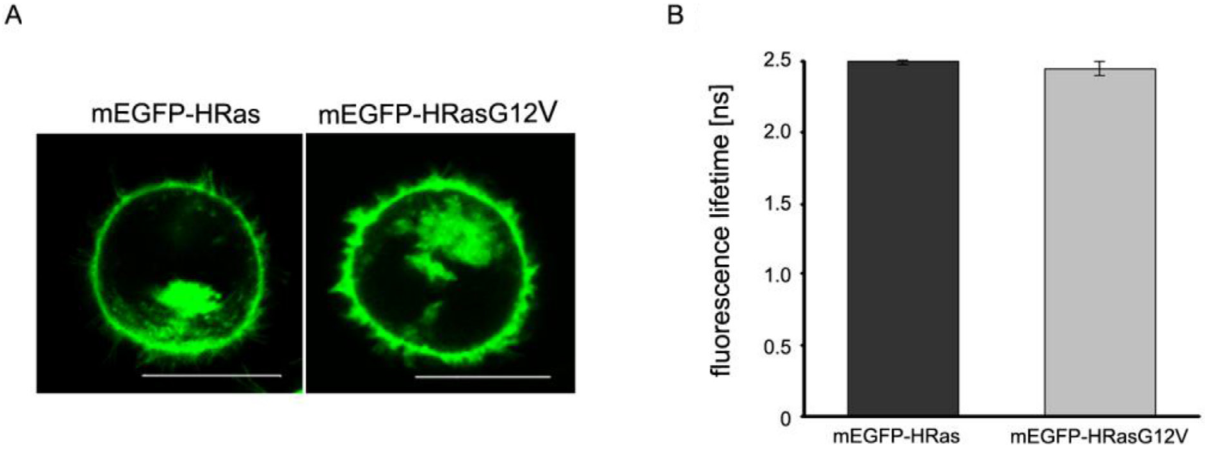

Supplementary Figure 3: (**A**) Representative confocal images of HEL cell overexpressing mEGFP-HRas and mEGFP-HRasG12V (G12V). (**B**) Mean fluorescence lifetime values ± S.D. of mEGFP-HRas and mEGFP-G12V. Scale bars 10 μm.

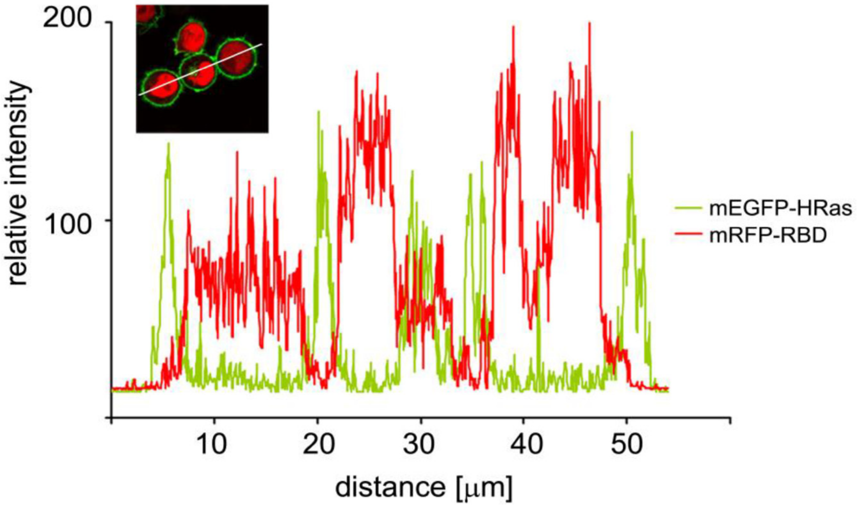

Supplementary Figure 4: Representative relative fluorescence intensity profile of analyzed HEL cells overexpressing mEGFP-HRas and mRFP-RBD.

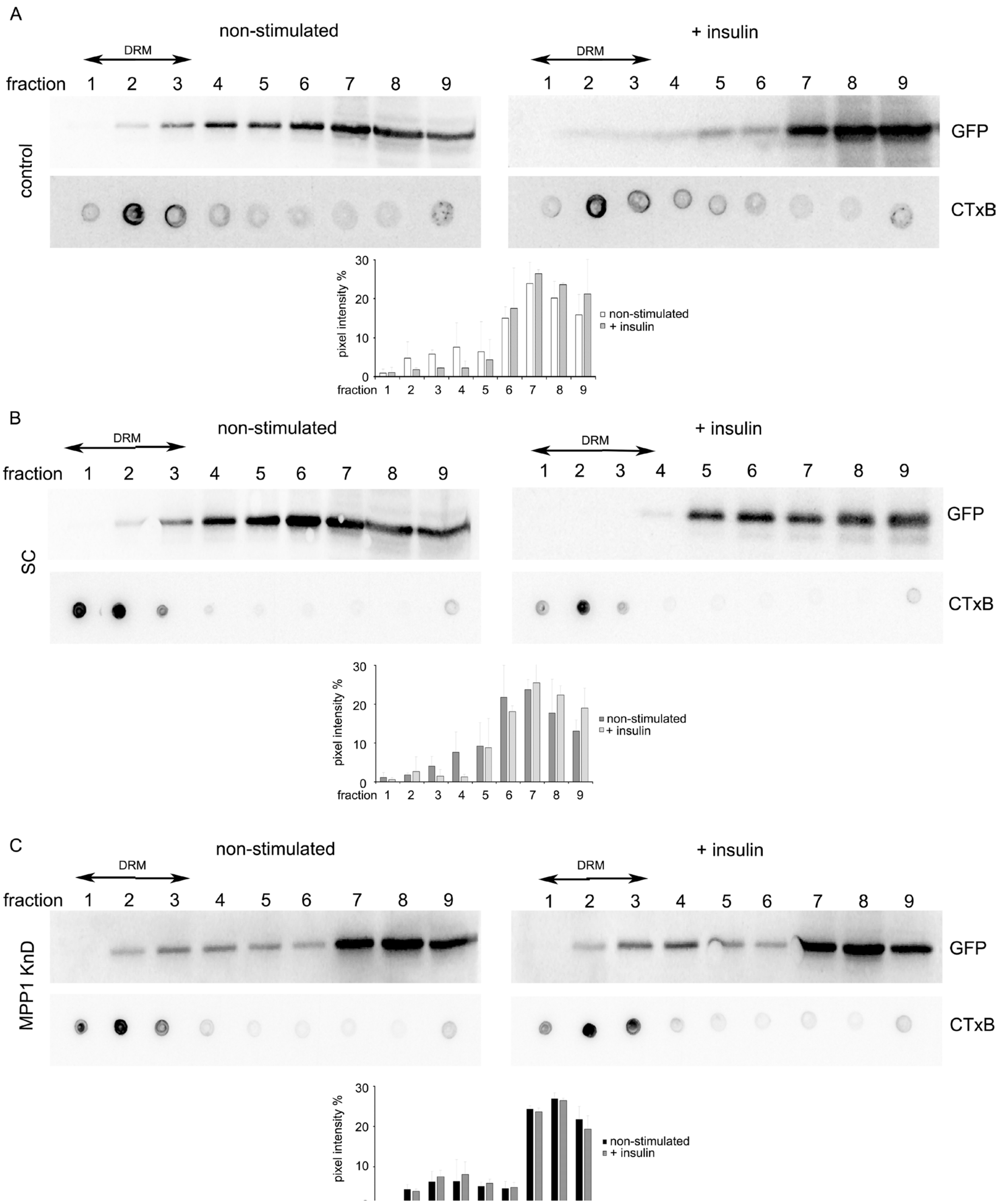

Supplementary Figure 5: mEGFP-HRas transfected (**A**) control (**B**) SC and (**C**) MPP1 KnD cells were treated or not treated, with insulin [1 µg/ml] for 5 min, after which DRM isolation in a sucrose gradient was performed. Nine fractions were collected from the top of the tube and analyzed by Western Blot with the indicated antibodies. Cholera toxin β (CTxB) staining was used as a DRM marker. Representative immunoblotting data from three independent experiments are presented. Error bars represent S.D.

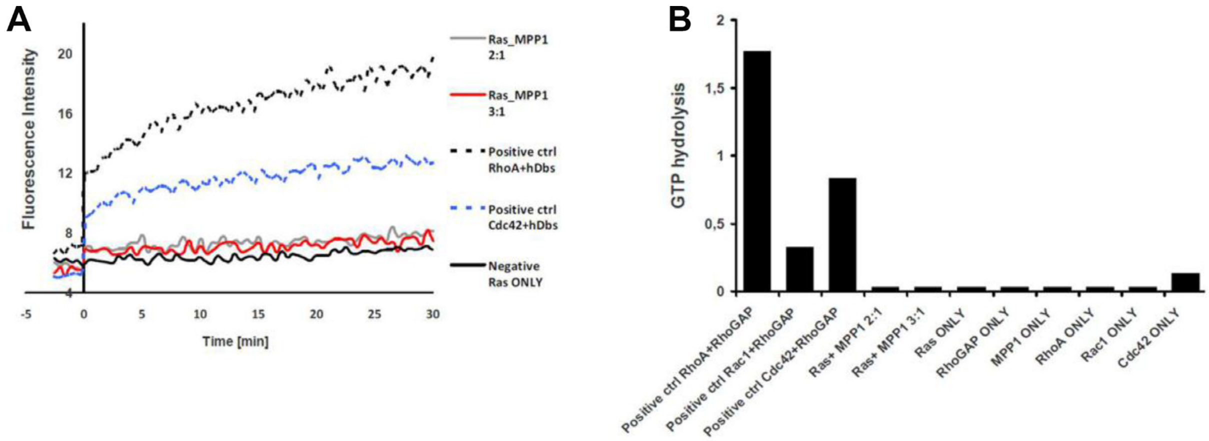

Supplementary Figure 6: (**A**) Recombinant MPP1 protein was used to check its potential GEF activity toward the Ras-GTPase in *in vitro* assay. MPP1 was incubated with His-HRas (2:1 or 3:1 ratio) in mixtures containing *N*-methylanthraniloyl (mant)-GTP and its GEF activity was determined by spectrofluorimetry. (**B**) Potential GAP activity of MPP1 toward Ras was assayed by *in vitro* RhoGAP assay. Different ratio of recombinant MPP1 and His-HRas protein were incubated in the presence or absence of RhoGAP domain. GAP activity was monitored by the measurement of free phosphate level generated by GTP hydrolysis.

Supplementary Table 1: FLIM-FRET analysis of interactions between mEGFP-HRas and mRFP-RBD

cells	FRET combination	*n*	τ_{av} [ns]	τ_D [ns]	τ_{DA} [ns]	f_{DA}	x^2
	mEGFP-HRas alone	10	2.50 ± 0.01	2.50	-	-	1.02 ± 0.05
control	mEGFP-HRasG12V alone	10	2.47 ± 0.02	2.47	-	-	0.99 ± 0.06
	mEGFP-HRasG12V + mRFP-RBD	10	1.94 ± 0.08	2.50[#]	0.92 ± 0.10	0.34 ± 0.03	1.04 ± 0.05
control	mEGFP-HRas + mRFP-RBD	31	2.49 ± 0.06	2.49 ± 0.06	-[*]	-	0.99 ± 0.05
control +insulin	mEGFP-HRas + mRFP-RBD	22	2.39 ± 0.05	2.50[#]	1.19 ± 0.38	0.12 ± 0.08	1.04 ± 0.06
SC	mEGFP-HRas + mRFP-RBD	27	2.47 ± 0.03	2.47 ± 0.03	-[*]	-	0.99 ± 0.06
SC +insulin	mEGFP-HRas + mRFP-RBD	22	2.36 ± 0.09	2.50[#]	1.28 ± 0.24	0.12 ± 0.05	1.05 ± 0.05
MPP1 KnD	mEGFP-HRas + mRFP-RBD	26	2.48 ± 0.04	2.48 ± 0.04	-[*]	-	1.04 ± 0.06
MPP1 KnD +insulin	mEGFP-HRas + mRFP-RBD	21	2.48 ± 0.04	2.50[#]	1.04 ± 0.04	0.01 ± 0.02	1.04 ± 0.05

[*]decays were successfully fitted with monoexponetial model, no donor-acceptor interaction observed.

[#]lifetime of donor was fixed at 2.50 ns which is the value obtained for mEGFP-HRas alone.

Abbreviations used: n – number of examined cells; τ_{av} – average fluorescence lifetime ± S.E.M; τ_D – fluorescence lifetime of the non-interacting donor fraction ± S.E.M; τ_{DA} – fluorescence lifetime of the interacting donor-acceptor fraction ± S.E.M; f_{DA} – the interacting donor fraction ± S.E.M; mEGFP – monomeric enhanced green fluorescent protein; mRFP – monomeric red fluorescent protein; RBD – Ras binding domain.
